# Newborn Care Practice and Associated Factors among Mothers of One-Month-Old Infants in Southwest Ethiopia

**DOI:** 10.1155/2020/3897427

**Published:** 2020-10-20

**Authors:** Amanuel Nuramo Sakelo, Nega Assefa, Lemessa Oljira, Zebene Mekonnen Assefa

**Affiliations:** ^1^Department of Midwifery, College of Medicine and Health Science, Wachamo University, Ethiopia; ^2^Department of Public Health, College of Health and Medical Science, Haramaya University, Ethiopia; ^3^Department of Nursing, College of Medicine and Health Science, Wolkite University, Ethiopia

## Abstract

Newborn care refers to the care that is provided to the baby from birth to one-month-old by a caregiver or by the mothers including thermal care, hygienic care, cord care, eye care, breastfeeding, immunization, and identification of newborn danger signs. According to Ethiopian Demographic and Health Survey (EDHS) 2016, the neonatal mortality rate was 29 deaths per 1000 live births, and the postneonatal mortality rate was 19 deaths per 1000 live births with neonates contributing 48 deaths per 1000 of the infant mortality. Neonatal mortality accounts for approximately two-thirds of all infant mortality worldwide. *Objective*. The objective of this study was to assess newborn care practice and associated factors among mothers with babies of one-month-old in Hossana town, Southern Nations, Nationalities, and Peoples' Region, Ethiopia, 2018. *Methods*. A community-based cross-sectional study was conducted among randomly selected 422 mothers with babies of one-month-old in Hossana town, southwest Ethiopia. The data were entered to EpiData 3.1 and exported to Statistical Package for the Social Sciences (SPSS) version 22. Bivariate and multivariate analyses were applied, and frequencies and odds ratios were calculated to determine the prevalence and associated factors, respectively. *Results*. In this study, 31% of participants had good newborn care practice based on three composite variables such as 84% who have done early breastfeeding initiation, 32.9% who have done safe cord care, and 30.6% who have done thermal care. Educational status of the mother's, primary (AOR = 2.80, 95% CI: 1.027-7.637), secondary (AOR = 2.596, 95% CI: 0.921-7.316), and college and above (AOR = 3.63, 95% CI: 1.056-12.492); mothers who practiced handwashing (hygiene) before touching a newborn (AOR = 2.552, 95% CI: 1.092-5.963); and mothers who had good knowledge on newborn care practice (AOR = 15.638, 95% CI: 3.599-67.943) were significantly associated with newborn care practice. *Conclusion and Recommendation*. The present study indicated that the level of comprehensive newborn care practice was unsatisfactory; all responsible bodies were giving attention and intervene on the predictors to improve newborn care practice and provide health education regarding newborn care practice. Education level, health education (counseling) on hygiene, and knowledge of mother on newborn care practice were independent predictors of newborn care practice.

## 1. Introduction

Essential newborn care is the basic care required for every baby and comprises thermal care (delayed bathing, drying, and keeping the baby warm through skin-to-skin contact), infection prevention (promoting and supporting handwashing for all caregivers and providing hygienic umbilical cord and skin care), feeding support (early and exclusive breastfeeding), and postnatal care, including monitoring of newborns for danger signs of serious infections and identifying babies requiring additional care [1]. Deaths in the newborn period (first 28 days) are a growing proportion of all child deaths [2], and essential newborn care practice is used to decrease neonatal morbidity and mortalities if given appropriately [3].

There is a global underfive mortality rate of 42.5 per 1000 live births; of those deaths, 45% were newborns, with a neonatal mortality rate of 19 per 1000 live births [4, 5], and although underfive and infant mortalities have been reduced, neonatal mortality remains largely unchanged in Nepal [6].

In Ethiopia, neonatal morbidity and mortality rates were among the highest in the world [7] and neonatal mortality was found to be 214 out of 4888 live births with the rate of 43.8 per 1000 live births in north Gonder [8], and also, according to the EDHS report, neonatal mortality was 29 per 1000, 41per 1000, and 38 per 1000 in urban and rural, respectively, in Ethiopia [9].

Even only 26% of births occur in a health facility, there is an increase in neonatal death [9]. To reduce newborn death, newborn care becomes the health priority [5]. Families, are focus on immediate newborn care at home and changes in household level practices to prevent newborn death, illness and to promote health of newborn care in Ethiopia [1]. Merely 13% of newborns receive a postnatal check within two days of birth [9]. The level of newborn care practice is scanty, inconclusive, and there are limited studies conducted in this area that focus on practices of newborn care and associated factors among mothers in this region (SNNPR).

## 2. General Objective

The objective of this study was to assess newborn care practice and associated factors among mothers of one-month-old infants in Hossana town, Hadiya zone, southern Ethiopia, 2018.

## 3. Specific Objectives

- To assess newborn care practice among mothers with babies aged one month
- To identify factors associated with newborn care practice among mothers with babies aged one month

## 4. Methods and Materials

### 4.1. Study Area and Period

Hossana town is the capital city of Hadiya zone, Southern Nations, Nationalities, and Peoples' Region (SNNPR), which was located 194 km from Hawassa, the capital city of the region, and 230 km from Addis Ababa, the capital city of the country.

Hossana town is a purely woinedega agroeconomic zone, situated at an altitude of 1800-2950 meters above sea level, and has an average temperature ranging from 10 to 24 degree centigrade. The annual rainfall is 1250 mm per year.

Based on the 2007 Ethiopian national population and housing census, the population of the town was 78,432: male 38,800 and female 39,632; the number of childbearing age women (15-49 years) was 18,275 (Hossana town administrative office report 2007).

A community-based cross-sectional study was conducted from January 20 to February 19, 2018, in Hossana town, southwest Ethiopia.

#### 4.1.1. Study Design

A community-based cross-sectional study design was conducted.

#### 4.1.2. Source of Population

The source population is all women in the reproductive age group, who had one-month age infants in Hossana town.

#### 4.1.3. Study Population

Study populations were all sampled mothers who had one-month age infants during the data collection period in Hossana town.

### 4.2. Inclusion and Exclusion Criteria

#### 4.2.1. Inclusion Criteria

Mothers resident in the area for six or more months before this study was conducted were included in the study.

#### 4.2.2. Exclusion Criteria

Mothers who were unable to feed breast milk and too sick or critically ill during the data collection period were excluded in the study.

### 4.3. Sample Size Determination

The sample size was determined by using a single population proportion formula:

(1) $$\begin{array}{c} n=\frac{{\left(Za/2\right)}^{2}p\left(1-p\right)}{D^{2}}, \end{array}$$

where *n* is the minimum sample, *p* is the 52.1% prevalence level for early breastfeeding on four regions of Ethiopia [10], *D* is the margin error (0.05), and *Z*(*a*/2) is the standard normal.

(2) $$\begin{array}{c} n=\frac{{\left(1.96\right)}^{2}x0.521\left(1-0.521\right)}{{\left(0.05\right)}^{2}}=384. \end{array}$$

Then, to get the final sample size (*N*), the nonresponse rate (10%) was used, 384 × 0.1 = 38.4 + 384 = 422.

### 4.4. Sampling Procedure

A survey was conducted among 422 women having a one-month infant in Hossana town. A multistage sampling technique was used to select study participants, and the study was conducted in all kebeles found in the town. A proportional allocation to the size of the population was done to decide the number of women required from each kebeles.

Finally, a simple random sampling technique was applied to identify women to be included in the survey. When more than one eligible respondent is present in a selected household, one respondent is selected on the spot by a lottery method.

## 5. Variables

### 5.1. Dependent Variable

- Newborn care practice (early breastfeeding, thermal care, and cord care)

### 5.2. Independent Variable

- Sociodemographic and socioeconomic factors (parity, occupation, education status, and place of delivery)
- Mothers' knowledge on newborn care practice (knowledge on newborn care practice and knowledge on newborn danger signs)
- Health service and obstetric factors (attendance of ANC, health education during ANC and PNC, health extension worker home visit, and neonatal death)

### 5.3. Data Collection Procedures (Instruments, Personnel, and Data Quality Control)

#### 5.3.1. Data Collection Instruments

The data were collected using a structured interviewer-administered questionnaire adapted from the EDHS, and other relevant literatures were used to collect data. The questionnaire had included all the questions that assess the knowledge and practice of newborn care of mothers. The tool was prepared in English version and translated to Hadiyisa (local language) and then translated back to English language to check for consistency. Finally, the data were collected by Hadiyisa language.

#### 5.3.2. Data Collection Process

Seven diploma midwives and five diploma nurses were recruited from other kebeles. Training was given for both data collectors and supervisors for two days before the actual data collection about data collection techniques go through the questionnaire questions with questions, ways of data collection, supervision and final clarification was given to those who have doubts.

### 5.4. Operational Definitions and Definition of Terms

Newborn care: it refers to the care provided to the baby from birth to 28 days of age by a caregiver or by the mothers including thermal care, hygienic care, cord care, eye care, breastfeeding, immunization, and identification of newborn danger signs.

Practice of newborn care: a mother was asked questions that cover the practice of newborn care which includes early initiation of breastfeeding and providing colostrums, cord care, and thermal care. The investigator developed composite index questions in the above issues that assigned a score of one [1] = correct response (consistent with the WHO essential newborn care guidelines) and 0 = incorrect response (inconsistent with the WHO/Unicef essential newborn care guidelines); any mother who did not know the answer is considered to have an incorrect response.

Good knowledge of mothers on newborn care: those mothers who respond correctly above 50% of knowledge-related questions.

Poor knowledge of mothers on newborn care: those mothers who respond correctly less than or equal to 50% of knowledge-related questions.

Newborn care practice: good newborn care practice: those mothers who mentioned three newborn care practices; poor newborn care practice: those who reported two or less newborn care practices.

Kebele: it is the smallest administrative unit, similar to a ward, a neighborhood, or a localized and delimited group of people and a part of woreda (district).

### 5.5. Data Quality Management

Data quality was assured by using a pretested data collection tool, and training was given for data collectors and supervisors before actual data collection. Supervisors were engaged in continuous supervision and monitoring during data collection. Completeness and consistency of data were checked by supervisors, data clerks, and investigators before and during data entry.

### 5.6. Data Analysis

Collected data was checked for its completeness and then coded and entered into EpiData version 3.1, and entered data was exported to SPSS version 20 for analysis. Binary and multivariate logistic regressions were employed. Frequencies and proportions were computed. A significant association was determined by odds ratios with *p* value < 0.05, at 95% confidence interval. Finally, the results were presented in the form of tables, figures, and charts as appropriate.

## 6. Results

A total of 422 mothers who had one-month infants were involved in the study, yielding a 100% response rate. The majority of respondents, 289 (68.5%), were between 25 and 34 years of age, 213 (50.5%) were housewives, 418 (99.1%) were married, 258 (61.1%) were protestant religion, 279 (66.1%) were Hadiya ethnicity, 378 (89.6%) had a formal education, and 46 (10.9%) had a high income (Table 1).

### 6.1. Health Care Service Utilization and Obstetric Information

From the participants, 303 (71.8%) had a home visit in the last one month and had health educations; 369 (87.4%) had knowledge on handwashing with soap and clean water before handling their neonate, 347 (82.2%) on keeping neonate dry and wrapping after delivery, 402 (95.3%) on breastfeeding immediately after birth within an hour, 383 (90.8%) on danger sign, 373 (88.4%) on immunization, and 361 (85.5%) on how to care for low-birth-weight baby by HEW.

The majority of participants, 351 (83.2%), had no history of neonatal death before this delivery, 411 (97.4%) had ANC follow-up when they were pregnant, and 405 (96.0%) had given birth at health facilities (Table 2).

### 6.2. Initiation of Breastfeeding, Cord Care, and Thermal Care Practice

From the total participants, 409 (96.9%) gave the first breast milk for their baby, 352 (85.4%) initiated breast milk within an hour after birth, 336 (79.6%) did not apply anything on the cord of the newborn baby, 214 (50.7%) took care of bleeding to keep the cord clean and safe, 144 (34.1%) kept the cord dry and clean to keep the cord clean and safe, and 64 (15.2%) took the newborn baby to a health facility in order to keep umbilical cord clean and safe.

More than half of the respondents, 215 (50.9%), had given the first bath for newborn baby within the first 24 hours of delivery, and 293 (69.4%) placed the newborn baby to skin-to-skin contact always until the baby becomes stable (Table 3).

### 6.3. Mothers' Knowledge on Newborn Care and Danger Signs

Among the 422 study participants, 406 (96.2%) had known about care for their newborn, 331 (78.4%) applied nothing to the cord immediately after cutting up to 7 days except ordered medication, 294 (69.7%) handled umbilical cord after cutting without dressing, 327 (77.5%) bathed her newborn baby after 24 hours after delivery, 372 (88.2%) breastfed their baby within 1 hour after delivery, 368 (87.2%) believed feeding breast milk as the first food for a newborn baby after delivery, and 367 (87.0%) had knowledge about newborn danger sign (Table 4).

**Figure 1:**
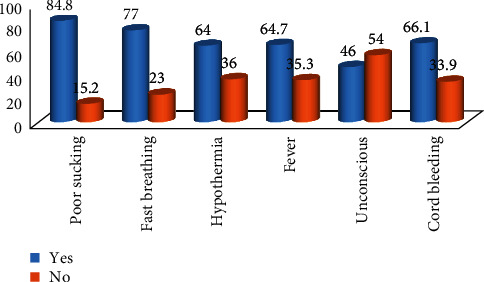
Identified newborn danger sign by mothers with babies of one-month-old after delivery in the community of Hossana town, southwest Ethiopia, 2018.

From the total of 422 participants, the mentioned danger signs of a newborn baby are as follows: 84.8% were poor sucking, 77% were fast breathing, 64% had hypothermia, 64.7% had fever, 46% had drowsiness (unconscious), and 66.1% had cord bleeding and infection (Figure 1).

**Figure 2:**
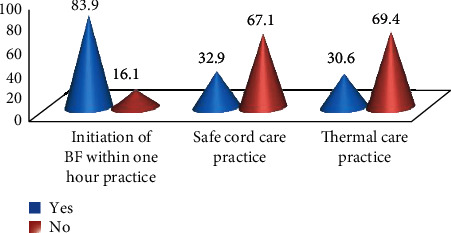
Identified three composite newborn care practices separately by mothers with babies of one-month-old after delivery in the community of Hossana town, southwest Ethiopia, 2018.

In this study, the proportion of newborn care practices was 130 (30.8%) of the respondents in terms of the three composite practices, namely, 354 (83.9%) were early breastfeeding initiation, 139 (32.9%) were safe cord care, and 129 (30.6%) were thermal care (delay bathing) (Figure 2).

### 6.4. Factors That Associated with Newborn Care Practice

In this study, education of the mothers, mothers who had practice handwashing, and knowledge of mothers on newborn care practice were significantly associated with newborn care practice.

Hence, those who had primary, secondary, and college and above educational status had three times (AOR = 2.80, 95% CI: 1.027-7.637), three times (AOR = 2.596, 95% CI: 0.921-7.316), and four times (AOR = 3.63, 95% CI: 1.056-12.492) more likely to practice newborn care than mothers who had no formal education, respectively, mothers who had practice handwashing were three times more likely to practice newborn care than mothers who had not practice handwashing (AOR = 2.552, 95% CI: 1.092-5.963), and mothers who had good knowledge on newborn care practice were sixteen times more likely to practice newborn care than those who had poor knowledge (AOR = 15.638, 95% CI: 3.599-67.943) (Table 5).

## 7. Discussion

In this study, one-third of the participants had good newborn care practice based on three composite variables such as early breastfeeding initiation 83.9%, safe cord care 32.9%, and thermal care 30.6%.

Good newborn care practice was almost nearly similar to study done in Aksum Town, North Ethiopia (26.7%) [11], but this study was lower than the study done in Mandura District, Northwest Ethiopia (40.6%), Gulomekada District, Eastern Tigray (92.9%), Mekelle City, North Ethiopia (81.1%) [7, 12, 13], and Damot pulasa Woreda, southern Ethiopia (24%) [14], and the difference may be due to socioeconomic, access of awareness among the study participants, geographical variation, and health-seeking behavior across the different cultures or cultural beliefs.

Breastfeeding 83.9% which was higher than study done in Hoima District, western Uganda 31% dry cord care 60.5% [15], Mandura District, Northwest Ethiopia (48.1%) [7], tamale metropolis of Ghana 70.5% [16], Tharu, Chitwan district 52.5% [6] and lower than study done in Mekelle City, North Ethiopia 97.4% [13], Aksum Town, North Ethiopia 63.1% [11].

Safe cord care was 32.9% and similar to the study done in Hoima District, western Uganda (31%) [15], but lower than the study done in Tharu, Chitwan district (95%) [6], Aksum Town, North Ethiopia (42.8%) [11], and Mewat, Haryana, India (49%) [17].

Thermal care was 30.6% in this study and nearly similar to the study done in Aksum Town, North Ethiopia (32.6%) [11], but lower than the study done in Hoima District, western Uganda (67.2%) [15], Mandura District, Northwest Ethiopia (37.8%) [7], Mekelle City, North Ethiopia (66.9%) [13], and Tharu, Chitwan district (96.6%) [6], and this difference may be due to relatively an increased awareness about the harmful effect of traditional foreign substance application to the umbilical cord.

Knowledge of the mother on newborn care practice had a significant association with newborn care practice and similar to the study in Hoima District, western Uganda, Gulomekada District, Eastern Tigray, and Mekelle City, North Ethiopia [12, 13, 15].

Education of the mothers (primary, secondary, and college and above) also has a significant association with newborn care practice and was similar to the study done in Mandura District, Northwest Ethiopia, and Mekelle City, North Ethiopia [7, 13].

Mothers who had health education on hygiene (hand) had a significant association with newborn care practice in this study, but no study was similar to this result.

## 8. Conclusions

In this study, almost one-third of the mothers had good newborn care practice and it was very low when compared with other studies done in the country. Mothers' educational status, mothers who had health education on hygiene, and knowledge of mothers on newborn care practice were independent predictors of newborn care practice.

## 9. Recommendations

Based on the findings of this study, we recommend the following:

- Hadiya Zone Health Bureau: to work hard on the promotion of health facility delivery system and to increase the level of newborn care practice and involve health extension workers to apply home-to-home visit program to convince all childbearing women on the positive outcome of health facility delivery service to have good newborn care practice
- Health care providers: to provide ongoing education and counseling to mothers to give birth at a health facility to have good newborn care practice during ANC follow-up
- Health care planners: to provide health education during ANC and PNC regarding these predictors
- Future researcher: we suggest researchers to undertake repeatable studies in this area, and as this study lacks qualitative information that can underpin the quantitative study results, we recommend that the researcher have to do qualitative study design and other methods

## 10. Limitation of the Study

The cross-sectional nature of the study is impossible to establish a temporal relationship between newborn care practice and identified risk factors.

## Figures and Tables

**Table 1:** Sociodemographic variables of mothers with babies of one-month-old in Hossana town, southwest Ethiopia, 2018 (*n* = 422).

Variables (*n* = 422)	Frequency (*n*)	Percent (%)
Age (year)		
15-24	76	18.0
25-34	289	68.5
≥35	57	13.5
Religion		
Protestant	258	61.1
Orthodox	106	25.1
Other	58	13.7
Ethnicity		
Hadiya	279	66.1
Kembata	60	14.2
Other	83	19.7
Marital status		
Married	418	99.1
Others	4	.9
Educational level		
Primary	137	32.5
College and above	129	30.6
Secondary	112	26.5
No formal education	44	10.4
Occupation		
Housewife	213	50.5
Governmental employee	117	27.7
Others	92	21.8
Wealth index		
Lowest	261	61.8
Middle	115	27.3
Highest	46	10.9

**Table 2:** Health care service utilization and obstetrics information of mothers with babies of one-month-old in Hossana town, southwest Ethiopia, 2018 (*n* = 422).

Variables (*n* = 422)	Frequency (*n*)	Percent (%)
HEW home visit		
Yes	303	71.8
No	119	28.2
Health education		
Yes	369	87.4
No	53	12.6
Keeping immediately dry and wrapping		
Yes	347	82.2
No	75	17.8
BF immediately within an hour		
Yes	402	95.3
No	20	4.7
HEW advice on danger signs		
Yes	383	90.8
No	39	9.2
HEW advice on immunization		
Yes	373	88.4
No	49	11.6
HEW advice on LBW care		
Yes	361	85.5
No	61	14.5
History of neonatal death		
Yes	71	16.8
No	351	83.2
ANC follow-up		
Yes	411	97.4
No	11	2.6
Place of current ANC visit		
Governmental health facility	376	89.1
Private health facility	46	10.9
Place of current delivery		
Home	17	4.0
Health facility	405	96.0

HEW = health extension workers; LBW = low birth weight; ANC = antenatal care.

**Table 3:** Practice of initiation of breastfeeding, cord care, and thermal care of mothers with babies of one-month-old in Hossana town, southwest Ethiopia, 2018 (*n* = 422).

Variables (*n* = 422)	Frequency (*n*)	Percent (%)
Did you give/feed colostrum		
Yes	409	96.9
No	13	3.1
When did you initiate breast milk		
Within an hour	352	85.4
After one hour	70	14.6
Did you apply anything on the cord		
Yes	86	20.4
No	336	79.6
What did you do to keep the cord safe		
Taking care of bleeding	214	50.7
Keeping it dry and clean	144	34.1
Taking to a health facility	64	15.2
When did you give a bath to baby		
Within 24 hours	215	50.9
After 24 hours	207	49.1
Was placed in skin-to-skin 1st		
Not at all	129	30.6
Always	293	69.4

**Table 4:** Knowledge on newborn care and danger signs of mothers with babies of one-month-old in Hossana town, southwest Ethiopia, 2018 (*n* = 422).

Variables (*n* = 422)	Frequency (*n*)	Percent (%)
Mother care to newborn baby		
Yes	406	96.2
No	16	3.8
What substance was applied to the cord		
Nothing applied	331	78.4
Butter applied	91	21.6
How long was the cord handled after cutting		
With dressing/cover	128	30.3
Without dressing	294	69.7
How long after birth was the baby washed for the 1^st^ time		
Within 24 hours	95	22.5
After 24 hours	327	77.5
How long after birth should the baby be breastfed		
Within one hour	372	88.2
After one hour	50	11.8
What should mother feed baby first		
Breast milk	368	87.2
Other than breast milk	54	12.8
Newborn danger sign		
Yes	367	87.0
No	55	13.0

**Table 5:** Factors that are associated with newborn care practice of mothers with babies of one-month-old in Hossana town, southwest Ethiopia, 2018.

Variables (*n* = 422)	Good practice of NBC	Poor practice of NBC	AORs (95% CI)	*p* value
Education level				
Primary	46 (33.6%)	91 (66.4%)	2.801 (1.027-7.637)	0.044^∗^
Secondary	33 (29.5%)	79 (70.5%)	2.596 (0.921-7.316)	0.071
College and above	46 (35.7%)	83 (64.3%)	3.633 (1.056-12.492)	0.041^∗^
No education	6 (13.6%)	38 (86.4%)	1	1
Health education (hand wash)				
Yes	123 (33.3%)	246 (66.7%)	2.552 (1.092-5.963)	0.030^∗^
No	8 (15.1%)	45 (84.9%)	1	1
Mothers' knowledge on newborn care practice				
Good	129 (35.4%)	235 (64.6%)	15.638 (3.599-67.943)	<0.001^∗^
Poor	2 (3.4%)	56 (96.6%)	1	1

^∗^Significant association.

## Data Availability

The authors confirm that the data supporting the findings of this study are available within the article and its supplementary materials.

## Abbreviations

ANC: Antenatal care
CSA: Central Statistical Agency
EDHS: Ethiopian Demographic and Health Survey
ENBC: Essential newborn care
HEW: Health extension worker
ICF: Infant and children feeding
IHRERC: Institutional Health Research Ethics Review Committee
PNC: Postnatal care
USAID: United States Agency for International Development
VDCs: Village Development Communities.
